# Screening and optimisation of in vitro pollen germination medium for sweetpotato (*Ipomoea batatas*)

**DOI:** 10.1186/s13007-023-01050-w

**Published:** 2023-08-29

**Authors:** Zongkuan Weng, Yitong Deng, Fen Tang, Lukuan Zhao, Lingxiao Zhao, Yuan Wang, Xibin Dai, Zhilin Zhou, Qinghe Cao

**Affiliations:** Xuzhou Institute of Agricultural Sciences in Jiangsu Xuhuai District/Institute of Sweetpotato Research, Chinese Academy of Agricultural Sciences, Xuzhou, 221121 China

**Keywords:** Pollen staining, Morphological structure, Viability detection, Temperature, Medium composition

## Abstract

**Background:**

Sweetpotato is an important vegetable and food crop that is bred through sexual crosses and systematic selection. The use of in vitro germination of sweetpotato pollen to test its viability has important theoretical and practical implications for improving the efficiency of sweetpotato crossbreeding by controlling pollination and conducting research on sweetpotato pollen biology.

**Results:**

In this study, we observed the morphological structure of sweetpotato pollen under a scanning electron microscope (SEM), developed an effective method for the in vitro germination of sweetpotato pollen, and examined the viability of sweetpotato pollen after treating plants at different temperatures before blossoming. Sweetpotato pollen grains are spherical, with an average diameter of 87.07 ± 3.27 μm (excluding spines), with multiple germination pores and reticulate pollen surface sculpture. We applied numerous media to sweetpotato pollen germination in vitro to screen the initial medium and optimised the medium components through single-factor design. The most effective liquid medium for in vitro sweetpotato pollen germination contained 50 g/L Sucrose, 50 g/L Polyethylene glycol 4000 (PEG4000), 100 mg/L Boric acid and 300 mg/L Calcium nitrate, with a pH = 6.0. The optimum growth temperature for pollen development in sweetpotato was from 25 to 30 °C. Neither staining nor in situ germination could accurately determine the viability of sweetpotato pollen.

**Conclusions:**

In vitro germination can be used to effectively determine sweetpotato pollen viability. The best liquid medium for in vitro germination of sweetpotato pollen contained 50 g/L Sucrose, 50 g/L Polyethylene glycol 4000 (PEG4000), 100 mg/L Boric acid and 300 mg/L Calcium nitrate, with the pH adjusted to 6.0. This study provides a reliable medium for the detection of sweetpotato pollen viability, which can provide a theoretical reference for sweetpotato genetics and breeding.

**Supplementary Information:**

The online version contains supplementary material available at 10.1186/s13007-023-01050-w.

## Introduction

Sweetpotato (*Ipomoea batatas* [L.] Lam.) is a nutrient-rich food and vegetable crop grown in most parts of the world due to its wide adaptability and high yields on marginal lands. Sweetpotato is used as a staple food in many developing countries, particularly in sub-Saharan Africa, including in Kenya, Uganda and Rwanda, where it is a major source of energy and nutrition for the local populations [1, 2]. Sweetpotato is rich in minerals, dietary fibre, antioxidants and β-carotene and has a higher nutritional and health value than other food crops [3–5]. Developing new sweetpotato varieties can enhance the performance of desirable agronomic traits in sweetpotato, such as improved carbohydrate content, enhanced resistance and higher nutrient content, to exploit the genetic potential of sweetpotato and meet the requirements of consumers [6–9].

Currently, sexual crosses and systematic selection are still the main methods used in sweetpotato breeding; relying on fertilisation between male and female gametes [10]. Fertilisation in angiosperms is an extremely complex process but can be simply described as a process whereby the paternal pollen lands on the stigma of the maternal flower and then grows a pollen tube, after which the pollen tube transports a pair of sperm through the style to the ovule, where it then fuses with the egg cell and central cell [11–13]. The highly cross-incompatible and self-incompatible characteristics of sweetpotato greatly increase the amount of work involved in and reduce the efficiency of crossbreeding, which greatly restricts the development of sweetpotato genetic breeding [10]. As key factors determining the success of crosses, pollen viability and interparental compatibility are also important reference indicators for parental selection. Therefore, the detection of sweetpotato pollen viability has important theoretical and practical significance for improving the efficiency of sweetpotato crossbreeding and conducting research on sweetpotato pollen biology.

The main methods used to test pollen viability are morphological observation, staining and in vitro germination [14]. The morphological observation method provides a preliminary determination of pollen activity based on structural changes in pollen morphology. This method is simple to use but does not accurately reflect the specific situation of pollen viability, making it only suitable as a reference for determining pollen viability. The staining method indicates pollen viability by producing a stable colour change when starch or enzymes in the pollen combine with a specific stain. The results of the staining method are more accurate than those of morphological observation. However, staining of the same pollen using different stains often shows different results. The in vitro germination method is based on the principle that pollen can germinate in a suitable medium. Pollen viability is determined through the preparation of a suitable medium and the assessment of its germination rate under suitable temperature and humidity conditions. This method is the preferred method for determining pollen viability in crossbreeding as it is accurate, reliable, practical and fully quantitative.

This study aimed to develop an optimised medium for the in vitro germination of sweetpotato pollen with the maximum germination rate. First, we completed the palynological information of sweetpotato by observing and describing the morphological structure of sweetpotato pollen under a scanning electron microscope (SEM). Second, we used numerous media for sweetpotato to screen the initial medium for optimisation and tested it in different genotypes. Third, we evaluated the effect of temperature changes on sweetpotato pollen viability on the day before flowering. Finally, we compared the effectiveness of different methods for measuring pollen viability.

## Results

### Pollen morphology under a SEM

**Fig. 1:**
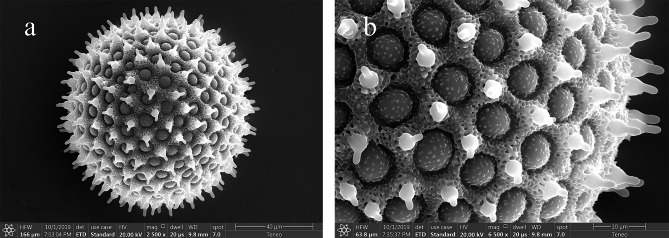
Micro-morphology of pollen grains of sweetpotato under a scanning electron microscope (SEM). **(a)** Overall view, bar = 40 μm; **(b)** exine sculptures, bar = 10 μm

The sweetpotato released pollen as monads, and the pollen grains were spherical, pantoporate and non-polar with an average diameter of 87.07 ± 3.27 μm (excluding spines) (Fig. 1a). The ornamentation of the sweetpotato pollen was reticulum. The average diameter of the pores was 6.81 ± 0.55 μm, with four to six spines surrounding each pore. The spines were 6.03 ± 0.31 μm long, tapering from base to tip with a blunt tip and a distinctly constricted neck. The bases of the adjacent spines were connected by reticulated ridges, and the bases of the spines and ridges were porous. The reticulation was small and suborbicular but irregular in shape (Fig. 1b).

### Screening of the initial medium

**Fig. 2:**
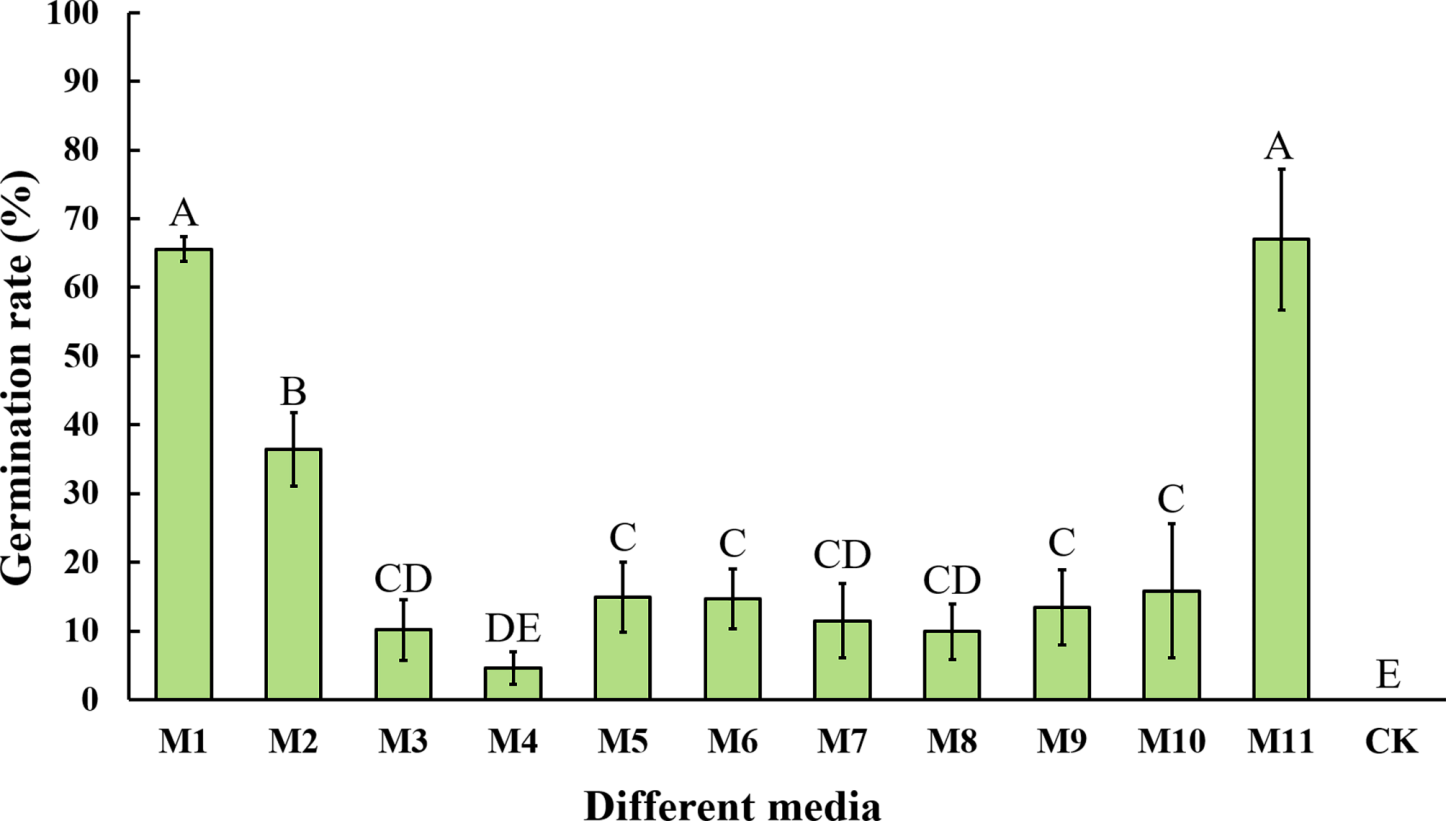
Pollen germination rate in response to different media. Different letters indicate statistically significant differences (*P* < 0.001), and error bars represent standard deviations

The average germination rate of sweetpotato pollen in the 11 media (Table 1) ranged from 4.63 to 66.98%, with no germination in pure water medium (Fig. 2). Through analysis of variance (ANOVA), the 11 different liquid media were found to have a significant effect on sweetpotato pollen germination (*P* < 0.001). The two media with the highest germination rates, M1 (65.6%) and M11 (67.0%), were significantly more effective in sweetpotato pollen germination than the other nine media. The germination rates between M1 and M11 were particularly close, with no significant differences; thus, the simpler composition of M11 was chosen as the initial medium for the subsequent optimisation of in vitro sweetpotato pollen germination.

**Table 1:** 11 different media for screening initial medium for sweetpotato pollen germination

Treatment	Plant species	Composition of culture medium								pH
		Sucrose (g/L)	Polyethylene glycol 4000 (PEG4000) (g/L)	Boric acid (mg/L)	Calcium nitrate (mg/L)	Calcium chloride (mg/L)	Potassium nitrate (mg/L)	Magnesium sulphate (mg/L)	GA3 (mg/L)	
M1	Wheat (Kanwal et al. 2022)	50	100	100	200		100	100		6.5
M2	Cherimoya (Lora et al. 2006)	80		100	250		100	200		
M3	Tomato (Karapanos et al. 2010)	100	151	50	300		100	200		
M4	Avocado (Alcaraz et al. 2011)	100	230	100	480		100	300		
M5	*Betula utilis* (Wani et al. 2020)	100		100	300		100	200		
M6	Feijoa (Xiong et al. 2016)	100		120.51		44.4			80	
M7	*Armeniaca sibirica* (Wu et al. 2022)	150		100	100					6
M8	*Exochorda racemosa* (Jia et al. 2022)	150		100	150				50	7
M9	Soybean (Salem et al. 2007)	150		100	300					
M10	Quinoa (Castillo et al. 2022)	160		300	70					5.5
M11		50	100	100	200					6.5
CK		0	0	0	0	0	0	0	0	6

### Optimisation of pollen germination media pH

After the initial medium was determined, the effects of five pH levels (4.5, 5.0, 5.5, 6.0 and 6.5) on pollen germination rates were assessed (Table 2). ANOVA showed that the germination rate was significantly higher at pH levels of 5.5, 6.0 and 6.5 than at pH levels of 4.5 and 5.0 (*P* < 0.001). There was no significant difference in germination rates between the three treatments with pH ≥ 5.5. Therefore, pH 5.5, 6.0 and 6.5 could all be used for optimal germination. In the present study, pH 6.0 was chosen for subsequent experiments, because the pH of the freshly configured medium was closest to this value.

**Table 2:** Media used for the pH test

Treatment	pH	Sucrose (g/L)	Polyethylene glycol 4000 (PEG4000) (g/L)	Boric acid (mg/L)	Calcium nitrate (mg/L)	Germination rate (%)
pH medium 1	4.5	50	100	100	200	24.1 ± 10.9
pH medium 2	5.0	50	100	100	200	30.1 ± 6.3
pH medium 3	5.5	50	100	100	200	52.4 ± 3.7*
pH medium 4	6.0	50	100	100	200	66.9 ± 4.7*
pH medium 5	6.5	50	100	100	200	53.6 ± 5.2*

Germination rate is the mean ± SD of five replicates. * Indicates the first echelon of multiple comparison results. ANOVA showed that the germination rate was significantly higher at pH 5.5, 6.0 and 6.5 than at 4.5 and 5.0 (*P* < 0.001)

### Optimisation of sucrose content in pollen germination media

After determining the optimum pH level of the initial medium, seven media with differing Sucrose concentrations (0, 50, 100, 150, 200, 250 and 300 g/L) were set up for in vitro sweetpotato pollen germination to determine the optimum Sucrose concentration (Table 3). The ANOVA results indicated that pollen germination rates differed significantly (*P* < 0.001) between the different Sucrose concentration treatments. With no Sucrose in the medium, the average germination rate was only 3.9%, and the highest germination rate was achieved at a Sucrose concentration of 50 g/L, with an average germination rate of 64.8%. The germination rate decreased with a gradual increase in Sucrose concentration and approached zero when the Sucrose concentration was 30 g/L. The Sucrose concentration was adjusted to 50 g/L in all subsequent experimental media.

**Table 3:** Media used for the Sucrose concentration test

Treatment	pH	Sucrose (g/L)	PEG4000 (g/L)	Boric acid (mg/L)	Calcium nitrate (mg/L)	Germination rate (%)
Sucrose medium 1	6.0	0	100	100	200	3.9 ± 2.0
Sucrose medium 2	6.0	50	100	100	200	64.8 ± 4.3*
Sucrose medium 3	6.0	100	100	100	200	31.9 ± 2.2
Sucrose medium 4	6.0	150	100	100	200	23.1 ± 3.0
Sucrose medium 5	6.0	200	100	100	200	6.0 ± 1.7
Sucrose medium 6	6.0	250	100	100	200	3.4 ± 1.2
Sucrose medium 7	6.0	300	100	100	200	0.7 ± 1.0

Germination rate is the mean ± SD of five replicates. * Indicates the first echelon of multiple comparison results. The ANOVA results indicated that pollen germination rates differed significantly (*P* < 0.001) between the different Sucrose concentration treatments

### Optimisation of the PEG4000 content of pollen germination media

In this study, the effects of seven PEG4000 concentrations (0, 50, 100, 150, 200, 250 and 300 g/L) on in vitro sweetpotato pollen germination were evaluated (Table 4). The ANOVA results showed that pollen germination rates differed significantly (*P* < 0.001) between treatments with different PEG concentrations. The average pollen germination rate was 46.4% when the medium did not contain PEG, while it was 73.5% when the PEG4000 content was 50 g/L. As the PEG concentration increased, pollen germination rates decreased significantly. The PEG4000 content of all subsequent experimental media was adjusted to 50 g/L.

**Table 4:** Media used for the Polyethylene glycol 4000 (PEG4000) concentration test

Treatment	pH	Sucrose (g/L)	PEG4000 (g/L)	Boric acid (mg/L)	Calcium nitrate (mg/L)	Germination rate (%)
PEG4000 medium 1	6.0	50	0	100	200	46.4 ± 7.8
PEG4000 medium 2	6.0	50	50	100	200	73.5 ± 5.0*
PEG4000 medium 3	6.0	50	100	100	200	62.2 ± 7.8*
PEG4000 medium 4	6.0	50	150	100	200	34.1 ± 12.8
PEG4000 medium 5	6.0	50	200	100	200	12.7 ± 2.1
PEG4000 medium 6	6.0	50	250	100	200	11.8 ± 3.0
PEG4000 medium 7	6.0	50	300	100	200	11.2 ± 2.8

Germination rate is the mean ± SD of five replicates. * Indicates the first echelon of multiple comparison results. The ANOVA results showed that pollen germination rates differed significantly (*P* < 0.001) between treatments with different PEG concentrations

### Optimisation of boric acid concentrations in pollen germination media

The effect of five Boric acid concentrations (0, 100, 200, 300 and 400 mg/L) on the germination rate of sweetpotato pollen was evaluated (Table 5). The highest germination rate of sweetpotato pollen was achieved with the addition of 100 mg/L Boric acid. The ANOVA results showed that the difference in pollen germination rate between the media with and without Boric acid was significant (*P* < 0.01), while the difference between the four media containing Boric acid was not significant. Since the highest germination rate was observed under 100 mg/L Boric acid treatment, subsequent experiments used Boric acid concentration of 100 mg/L in the medium.

**Table 5:** Media used for the Boric acid concentration test

Treatment	pH	Sucrose (g/L)	Polyethylene glycol 4000 (PEG4000) (g/L)	Boric acid (mg/L)	Calcium nitrate (mg/L)	Germination rate (%)
Boron medium 1	6.0	50	50	0	200	10.0 ± 3.2
Boron medium 2	6.0	50	50	100	200	64.3 ± 5.5*
Boron medium 3	6.0	50	50	200	200	73.8 ± 5.5*
Boron medium 4	6.0	50	50	300	200	63.9 ± 3.7*
Boron medium 5	6.0	50	50	400	200	47.7 ± 5.7*

Germination rate is the mean ± SD of five replicates. * Indicates the first echelon of multiple comparison results. The ANOVA results showed that the difference in pollen germination rate between the media with and without Boric acid was significant (*P* < 0.01), while the difference between the four media containing Boric acid was not significant

### Optimisation of calcium nitrate content in pollen germination media

In this study, the effect of five Calcium nitrate concentrations (0, 100, 200, 300 and 400 mg/L) on sweetpotato pollen germination was evaluated (Table 6). Sweetpotato pollen had the highest average germination rate in the medium with a Calcium nitrate concentration of 300 mg/L. Based on ANOVA, the mean germination rates differed significantly between the media supplemented with and without Calcium nitrate (*P* < 0.01), while the differences between the four different Calcium nitrate concentrations were not significant. Therefore, 100 mg/L, 200 mg/L, 300 mg/L and 400 mg/L of Calcium nitrate can be used for optimal germination. In this study, 300 mg/L of Calcium nitrate was used in subsequent experiments. Thus, the optimised medium composition for this study was 50 g/L Sucrose, 50 g/L PEG4000, 100 mg/L Boric acid and 300 mg/L Calcium nitrate, with a pH level of 6.0.

**Table 6:** Media used for the Calcium nitrate concentration test

Treatment	pH	Sucrose (g/L)	Polyethylene glycol 4000 (PEG4000) (g/L)	Boric acid (mg/L)	Calcium nitrate (mg/L)	Germination rate (%)
Calcium medium 1	6.0	50	50	100	0	7.9 ± 2.4
Calcium medium 2	6.0	50	50	100	100	61.7 ± 5.7*
Calcium medium 3	6.0	50	50	100	200	71.9 ± 4.1*
Calcium medium 4	6.0	50	50	100	300	79.7 ± 4.6*
Calcium medium 5	6.0	50	50	100	400	61.6 ± 9.4*

Germination rate is the mean ± SD of five replicates. * Indicates the first echelon of multiple comparison results. Based on ANOVA, the mean germination rates differed significantly between the media supplemented with and without Calcium nitrate (*P* < 0.01), while the differences between the four different Calcium nitrate concentrations were not significant

### Effect of temperature treatment on pollen viability

Pollen viability was examined under different treatments by subjecting each individual plant to different temperatures (15, 20, 25, 30, 35, 40 and 45 °C) to the individual plant separately, for 24 h until flowering (Table 7). ANOVA conducted on pollen germination showed that pollen germination rates differed significantly (*P* < 0.01) between the temperature treatments, with pollen under the 25 and 30 °C treatments showing significantly higher germination rates than the other treatments. The highest average pollen germination rate of 75.8% was recorded for the 30 °C treatment. When the temperature was below 30 °C, the germination rate increased with the temperature. When the temperature was above 30 °C, the pollen germination rate decreased significantly with the increase in temperature, dropping to 4.7% when the temperature rose to 45 °C.

**Table 7:** Different temperature treatments applied to plants 24 h before flowering

Treatment	Temperature (°C)	Germination rate (%)
Temp. 1	15	22.7 ± 3.9
Temp. 2	20	33.5 ± 1.6
Temp. 3	25	74.1 ± 4.3*
Temp. 4	30	75.8 ± 5.5*
Temp. 5	35	34.2 ± 6.5
Temp. 6	40	5.5 ± 3.2
Temp. 7	45	4.7 ± 2.2

Germination rate is the mean ± SD of five replicates. * Indicates the first echelon of multiple comparison results. ANOVA on pollen germination showed that pollen germination rates differed significantly (*P* < 0.01) between the temperature treatments, with pollen from the 25 and 30 °C treatments showing significantly higher germination rates than the other treatments

### In vitro pollen germination of different genotypes

**Fig. 3:**
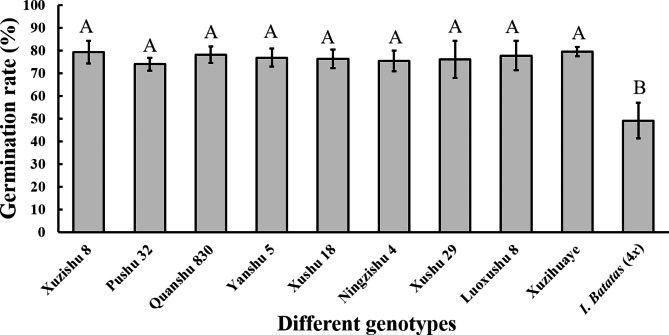
Pollen germination rates of different genotypes in response to the optimised medium. Different letters indicate statistically significant differences (*P* < 0.001), and error bars represent standard deviations

Pollen from 10 different sweetpotato genotypes was cultured in vitro in the medium containing 50 g/L Sucrose, 50 g/L PEG4000, 100 mg/L Boric acid and 300 mg/L Calcium nitrate, with pH = 6.0. ANOVA showed that the pollen germination rates were significantly different between cultivated sweetpotato and *I. batatas* (4*x*) (*P* < 0.001), while the differences between cultivated sweetpotato genotypes were not significant (Fig. 3). Among cultivated sweetpotato genotypes, Pushu 32 had the lowest germination rate (74.0%), and Xuzihuaye had the highest (79.6%). *Ipomoea batatas* (4*x*) had a germination rate of only 49.14%.

### Comparison of pollen viability testing methods

**Fig. 4:**
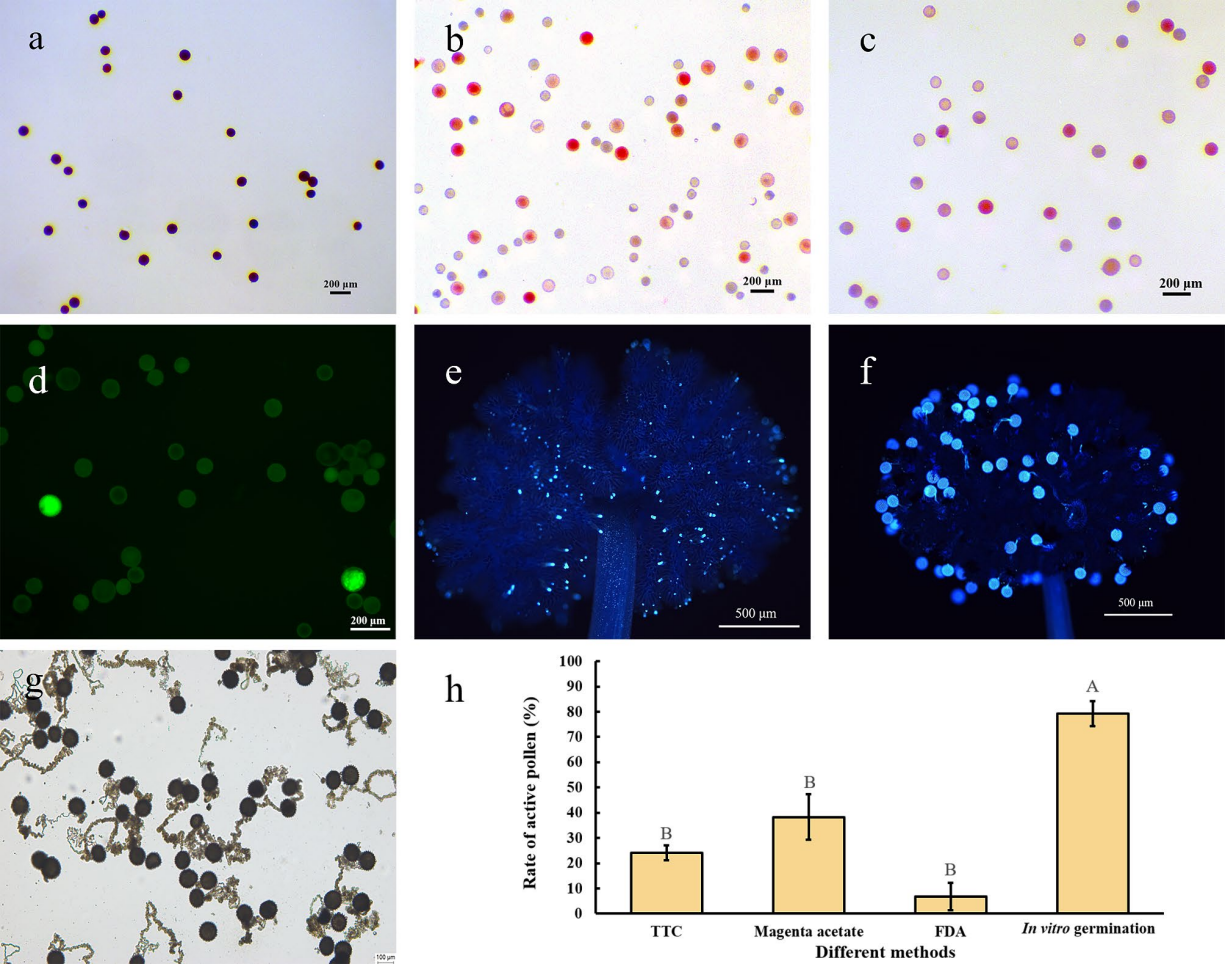
Comparison of different pollen viability testing methods. **(a)** I_2_-KI, bar = 200 μm. **(b)** TTC, bar = 200 μm. **(c)** Magenta acetate, bar = 200 μm. **(d)** FDA, bar = 200 μm. **(e, f)** In situ germination, bar = 500 μm. **(g)** In vitro germination, bar = 100 μm. **(h)** Active pollen rates measured by TTC, Magenta acetate, FDA and in vitro germination. Different letters indicate statistically significant differences (*P* < 0.001), and error bars represent standard deviations

For pollen staining, I_2_-KI stained all pollen blue (Fig. 4a), and TTC (Fig. 4b) and Magenta acetate (Fig. 4c) stained some of the pollen red. In the presence of FDA, active pollen showed green fluorescence (Fig. 4d). For in situ germination, Xuzishu 8 pollen germinated abundantly on the stigma of Pushu 32 (Fig. 4e) and did not germinate on its own stigma (Fig. 4f). The sweetpotato pollen germination rate in vitro reached nearly 80% (Fig. 4g). Pollen viability was significantly different (*P* < 0.01) when assayed by the three dyes (TTC, Magenta acetate and FDA) compared with in vitro pollen germination, while the differences between the different staining methods were not significant (Fig. 4h).

## Discussion

### Pollen morphological characteristics

As the carrier of the sperm cells of flowering plants, pollen is morphologically stable. Plants can be classified according to the morphological structure and surface ornamentation of the pollen. Changes in pollen morphology are also closely related to changes in pollen viability and can therefore be used as an important reference indicator for parental selection in crossbreeding. Srisuwan et al. [15] observed the morphological structure of pollen under an SEM in 10 *Ipomoea* spp. and found that all the pollen grains of these *Ipomoea* spp. were spherical and pantoporate, with four to six spines around each pore. The pollen grains of two sweetpotato genotypes, PI 318846 and PI 508520, were 97.9 ± 14.8 μm and 90.9 ± 4.1 μm in diameter, and the spines were 5 μm long. This is similar to the observations in this study, and the slight discrepancy, respectively, may be caused by the method of pollen treatment.

### Screening and optimisation of the initial medium

A reliable in vitro pollen germination medium allows for the accurate determination of pollen viability and is important for improving the practice and efficiency of crop genetic breeding and germplasm innovation. Different plants have different requirements for pollen germination media, but the components of the media are similar. Water is usually used as the solvent with certain solutes, such as Sucrose, PEG, Boric acid, Calcium nitrate, Magnesium sulphate and Potassium nitrate. Some plants even require the addition of plant growth hormones to promote pollen germination [14, 16–18].

In this study, ten pollen germination media applied in other plants were selected for sweetpotato pollen germination by pre-experimentation. The highest germination rate was obtained in the in vitro wheat pollen germination medium (Fig. 2). We simplified it by only using Sucrose, PEG4000, Boric acid and Calcium nitrate. The results showed that the germination rate of pollen in the simplified medium was not significantly different from that without simplification (Fig. 2).

The pH level of the medium is an important factor influencing the rate of in vitro pollen germination [19]. Setting a suitable pH level during in vitro pollen germination can improve the germination rate. Boavida and McCormick [20] reported that the germination rate of *Arabidopsis* pollen was only 50–60% in a medium at pH 7.0–7.5. When the pH level was increased to 7.5–8.0, the pollen germination rate increased to 70–80%. Burke et al. [21] found that the pH levels tested did not have a significant effect on cotton pollen germination rates in vitro. In our study, pollen germination rates were significantly higher at pH ≥ 5.5 than at pH ≤ 5.0 (Table 2). Rodriguez-Enriquez et al. [22] observed that germination rates increased with increasing pH and peaked at pH = 8.5 when performing in vitro pollen germination in *Arabidopsis thaliana*. In our results, there was no significant difference between the three treatments with pH ≥ 5.5, although germination rates peaked at pH = 6.0.

The addition of Sucrose and PEG to the medium can promote pollen germination [23]. Sucrose in the medium provides energy for pollen germination, while Sucrose also regulates the osmotic pressure of the solution together with PEG. PEG is an osmoregulator that is not metabolised by pollen, regulates the permeability of the plasma membrane and reduces pollen rupture [24]. Lin et al. [25] reported that an appropriate Sucrose concentration could promote pollen germination in oil palms, while an excessive Sucrose concentration inhibited pollen germination. In our experiments, the highest germination rates were obtained at a concentration of 50 g/L for both Sucrose and PEG tested separately (Tables 3 and 4), and the germination rates decreased with increasing concentrations above 50 g/L.

Boron is essential for pollen tube formation, and the addition of low concentrations of boron to the medium can promote pollen germination [26–28]. Obermeyer et al. [29] found that low concentrations of Boric acid stimulated H^+^-ATPase activity in the plasma membrane, accelerating ATP hydrolysis and H^+^ transport and thus promoting lily pollen germination and pollen tube formation. This is consistent with our results, where pollen germination rates were significantly higher in the four treatments to which Boric acid was added (Table 5) than in the treatment without Boric acid.

Calcium (Ca^2+^) is essential for pollen tube growth [30, 31]. Ca^2+^ regulates pollen tube elongation and orientation, and in vitro cultured pollen requires Ca^2+^ uptake from the medium [32]. This was well verified in our study, where the germination rate was below 10% without the addition of Ca^2+^ (Table 6), even though the medium was at optimum pH and the other ingredients were at optimum concentrations. For the treatments with added Calcium nitrate, the germination rate increased by over 60% in all cases.

### Effects of different temperature treatments on pollen viability

Plant pollen is highly sensitive to temperature stress, and even short-term temperature abnormalities during pollen development can result in reduced pollen viability [33, 34]. Cui et al. [35] reported that short-term low temperatures affect pollen development, leading to morphological and functional changes in pollen (including epidermal abnormalities, excessive starch accumulation, changes in pollen walls and excessive ROS production in anthers), thereby reducing pollen viability and fruit set. Masoomi-Aladizgeh et al. [36] found that although a temperature of 40 °C had little effect on plant photosynthesis during the short period of pollen development, pollen grains were significantly smaller and pollen viability was reduced by nearly 40% compared to pollen grown at normal temperatures (28 °C). Our research showed that changing the growing temperature of sweetpotato the day before flowering affected sweetpotato pollen activity (Table 7). Pollen viability was highest at 30 °C. Flowers were significantly smaller in diameter when the temperature was below 20 °C than at 25 °C. The flowers did not open properly at temperatures below 15 °C, and pollen viability decreased significantly above 30 °C.

### Comparison of different pollen viability testing methods

Staining is a common method for pollen viability identification and is based on a stable colour change resulting from the binding of starch or enzymes in the pollen to the stain [37]. When added to solutions of Iodine-postassium iodine (I_2_-KI), Tri-phenyl tetrazolium chloride (TTC), Magenta acetate and Fluorescein diacetate (FDA), the colour change extent of pollen can reflect pollen viability. Pollen staining is simple and rapid and can reflect the metabolism and nutritional content of pollen to some extent. However, it is influenced by the characteristics of the pollen itself (e.g., thickness of the pollen wall, composition of the outer pollen wall and strength of the various enzymatic activities within the pollen) and cannot accurately determine pollen viability. Alexander [38] used aniline blue staining and in vitro germination to determine the pollen viability of three *Hydrangea* L. plants and found that aniline blue staining greatly overestimated pollen viability compared to in vitro germination.

In our study, pollen viability measured by staining was significantly lower than that measured by in vitro germination (Fig. 4h). In addition, although in situ germination is closer to breeding practice, pollen germination on the stigma is also influenced by compatibility between parents. The number of pollen grains pollinated to the stigma was large, and the ungerminated pollen grains were shed during treatment (Fig. 4e), thus making it impossible to judge the actual viability of the pollen by the number of pollen grains that remained on the stigma and germinated.

## Conclusions

This study is the first to investigate the screening and optimisation for in vitro sweetpotato pollen germination and verifiy the utility of the medium using different sweetpotato genotypes. The in vitro germination rate of sweetpotato pollen was influenced by the concentration of various components of the culture medium. The best liquid medium for in vitro sweetpotato pollen germination contained 50 g/L Sucrose, 50 g/L PEG4000, 100 mg/L Boric acid and 300 mg/L Calcium nitrate, with the pH adjusted to 6.0. There was little difference in germination rates between cultivated sweetpotato genotypes. Pollen viability was easily affected by temperature, and the temperature suitable for sweetpotato flowering was 25–30 °C. I_2_-KI stained all pollen grains blue, making it impossible to determine whether pollen grains were viable. TTC, Magenta acetate or FDA could not be used to accurately determine pollen viability. This study provides an optimum medium composition for in vitro sweetpotato pollen germination, filling the gap in the in vitro detection of pollen viability in the field of sweetpotato breeding. It is of great significance for sweetpotato breeding and even for food security and seed industry revitalisation.

## Materials and methods

### Plant material and pollen collection

In this study, pollen from ten sweetpotato genotypes was used for the experiment: ‘Xuzishu 8’, ‘Pushu 32’, ‘Quanshu 830’, ‘Yanshu 5’, ‘Xushu 18’, ‘Ningzishu 4’, ‘Xushu 29’, ‘Luoxushu 8’, ‘Xuzihuaye’ and *I. batatas* (4*x*). The pollen of the easy-to-flower sweetpotato variety Xuzishu 8 was used to optimise the in vitro sweetpotato pollen germination medium, and the efficiency of the optimised medium was evaluated with the remaining nine genotypes. Healthy cuttings were selected, planted in sterilised soil and transferred to the insect screen room. After the plants were established and a certain extent of nutritional growth was completed, a dark treatment (8 h of light and 16 h of darkness per day) was applied to induce the flowering phase. When the plants were flowering, the anthers were removed from the freshly opened and normally developed flowers with forceps at approximately 8:30 am and placed in centrifuge tubes. About 1 mL of distilled water was added to the tubes. They were then centrifuged until the pollen grains were released from the anthers into the distilled water. When the pollen was settled to the bottom of the centrifuge tubes, the anthers and distilled water were removed, and the pollen was retained for subsequent experiments.

### SEM observation of pollen morphology

The fresh pollen grains were placed sequentially in different concentrations of alcohol solutions (25%, 50%, 70%, 80%, 90%, 95%) for 15 min to dehydrate. The extracted pollen grains were thoroughly dried in an oven at 40 °C for approximately 12 h and then glued to the SEM metal sample stage utilising double-sided copper conductive adhesive and placed in a high vacuum coater (Leica EM ACE600, Leica, Germany) for platinum spraying to cover the sample surface with a thickness of approximately 4 nm. Only a few pollen grains were scattered and adhered to the SEM metal sample stage to reduce pollen grain debris, impurities and pollen grain stacking from affecting the observation. The platinum-sprayed sample was observed using an SEM (Teneo Volume Scope, FEI, USA). The accelerating voltage of the SEM was set to 20 kV, the current to 50 pA and the working distance to 10 mm, which changed slightly during the focusing process. The pollen grains were located, and their surface morphology was observed. Photographs were taken in slow scanning mode after adjusting the magnification, and the pollen morphological characteristics were described, referring to a previous report [39].

### Screening of the initial medium

To screen a suitable initial medium formulation for in vitro sweetpotato pollen germination, extensive pre-experiments were conducted using many pollen germination media used in other plants. We screened 10 media used in different plants [40–49] (Table 1, M1–M10) and simplified the composition of M1 (Table 1, M11), which had the best germination efficiency in the pre-experiments. Pollen germination rates were compared between different media and pure water (Table 1, CK), and the medium with the highest germination rate was selected for optimisation as the initial medium for in vitro sweetpotato pollen germination.

### Pollen germination and data analysis

All of the components of each medium were dissolved in distilled water (all reagents used in this study were analytical reagents), and the pH was adjusted with 1 M NaOH and 1 mM HCl. The configured liquid medium was sterilised by placing it in ultraviolet light for 30 min. A total of 100 µL of liquid medium was pipetted into a centrifuge tube containing approximately 25 mg pollen, and the pollen was suspended and dispersed in the liquid medium. The liquid medium containing the pollen was immediately transferred to a slide and incubated at a constant temperature of 25 °C and humidity of 70% for at least 2 h. Five replicates of each medium treatment were set up. At the end of the culture, slides containing the medium and pollen were placed under a microscope (Leica DM4000 B, Leica, Germany) for observation, and images were taken of a field of view where the number of pollen grains was more than 30. Pollen tubes longer than the pollen grain diameter were considered germinated, and pollen germination rates were counted using Image J (version 1.51j8). The data were analysed with analysis of variance (ANOVA) using R (version 4.2.2). The pollen germination rate was calculated using the following formula:

$$\begin{gathered}{\text{Germination rate }}(\% ){\text{ = }} \hfill \\\frac{{{\text{Number of germinated pollen grains}}}}{{{\text{Total number of pollen grains}}}} \times {\text{100}} \hfill \\\end{gathered}$$

### Germination medium optimisation

After determining the initial medium for in vitro sweetpotato pollen germination, the pH, Sucrose, Polyethylene glycol 4000 (PEG4000), Boric acid and Calcium nitrate concentrations of the initial medium were optimised by conducting separate single-factor experiments. The optimum levels of pH (4.5, 5.0, 5.5, 6.0 and 6.5), Sucrose (0, 50, 100, 150, 200, 250 and 300 g/L), PEG4000 (0, 50, 100, 150, 200, 250 and 300 g/L), Boric acid (0, 100, 200, 300 and 400 mg/L), and Calcium nitrate (0, 100, 200, 300 and 400 mg/L) were explored so that the initial medium was progressively optimised.

### Evaluation of the effects of temperature on pollen germination

To investigate the effects of short-term external temperature changes on sweetpotato pollen viability before flower opening, Xuzishu 8 was chosen as the experimental material. Different temperatures (20, 25, 30, 35, 40 and 45 °C) were applied to the blooms 24 h before flowering until flowering, and pollen viability was tested at 8:30 am on the day of flowering using the optimised liquid medium (50 g/L Sucrose, 50 g/L PEG4000, 100 mg/L Boric acid and 300 mg/L Calcium nitrate, with pH = 6.0) at 25 °C.

### Evaluation of pollen germination rates of different genotypes in an optimised medium

To test the effect of different sweetpotato genotypes on the optimised pollen germination medium, nine genotypes (Xuzishu 8, Pushu 32, Quanshu 830, Yanshu 5, Xushu 18, Ningzishu 4, Xushu 29, Luoxushu 8 and Xuzihuaye) and *I. batatas* (4*x*) were selected for in vitro pollen germination. Five replicates of each genotype were used to compare the pollen germination rates of different genotypes.

### Comparison of different pollen viability testing methods

To compare the accuracy of different methods for testing pollen viability, pollen germination in situ was performed, and four stains were selected, Iodine-potassium iodide (I_2_-KI), Tri-phenyl tetrazolium chloride (TTC), Magenta acetate and Fluorescein diacetate (FDA) for pollen staining. For in situ pollen germination, the stamens were removed the day before the maternal flowers opened, and the flowers were covered with insect-proof paper bags. The next day, after the flowers had opened, the pollen from Xuzishu 8 was pollinated onto the stigma of the female (here, two genotypes were chosen: Xuzishu 8 and Pushu 32). The pollinated female flowers were then kept under the bags for 2 h. after which the pistils were collected, immersed in Carnot fixative and stained with Aniline Blue after 24 h. When staining, the stain was added to the centrifuge tube containing the pollen for 5–20 min, after which the stain was removed. The pollen was washed two or three times with distilled water, and the pollen was transferred to a slide for observation under a stereomicroscope (Leica S6 D, Leica, Germany). For FDA staining, the pollen was aspirated onto a slide for observation under a fluorescence microscope (Leica DM4000 B, Leica, Germany) after the staining solution was added.

### Electronic supplementary material

Below is the link to the electronic supplementary material.

Supplementary Material 1

## Data Availability

All data generated or analyzed during this study are included in this published article [and its supplementary information files].
