# Supplementary material for: Screening and optimisation of in vitro pollen germination medium for sweetpotato (Ipomoea batatas)
Source: Plant Methods. 2023 Aug 29;19:93. doi: 10.1186/s13007-023-01050-w (PMC10463589; doi:10.1186/s13007-023-01050-w)
Supplement: Supplementary file 1 — Supplementary Material 1 [file 13007_2023_1050_MOESM1_ESM.docx]

Table S1. Pollen germination rate in response to different media

| Treatments | Germination rate (%) | | | | |
| --- | --- | --- | --- | --- | --- |
| M1 | 63.16 | 65.96 | 64.38 | 66.67 | 67.65 |
| M2 | 36.96 | 30.91 | 42.55 | 40.54 | 30.99 |
| M3 | 5.88 | 12.82 | 9.52 | 6.25 | 16.22 |
| M4 | 6.25 | 1.94 | 6.38 | 2.13 | 6.45 |
| M5 | 15.71 | 22.73 | 15.00 | 12.12 | 9.09 |
| M6 | 21.57 | 10.94 | 15.63 | 13.89 | 11.11 |
| M7 | 2.63 | 10.20 | 15.91 | 13.33 | 15.22 |
| M8 | 5.77 | 8.33 | 14.71 | 6.98 | 13.73 |
| M9 | 9.62 | 16.67 | 8.93 | 10.42 | 21.57 |
| M10 | 8.00 | 3.92 | 20.83 | 28.00 | 18.37 |
| M11 | 54.00 | 62.50 | 73.53 | 64.44 | 80.43 |
| CK | 0.00 | 0.00 | 0.00 | 0.00 | 0.00 |

Table S 2. Pollen germination rate under different pH levels of media

| Treatment | Germination rate (%) | | | | |
| --- | --- | --- | --- | --- | --- |
| 4.5 | 38.10 | 32.43 | 14.63 | 22.22 | 13.33 |
| 5.0 | 19.61 | 32.43 | 32.73 | 29.69 | 36.23 |
| 5.5 | 54.55 | 53.49 | 50.00 | 47.37 | 56.67 |
| 6.0 | 70.97 | 72.55 | 65.12 | 61.36 | 64.29 |
| 6.5 | 50.00 | 60.61 | 51.43 | 47.06 | 55.32 |

Table S 3. Pollen germination rate under different Sucrose concentration media

| Concentrations (g/L) | Germination rate (%) | | | | |
| --- | --- | --- | --- | --- | --- |
| 0 | 5.13 | 3.81 | 4.00 | 4.41 | 2.13 |
| 50 | 64.52 | 60.42 | 67.27 | 61.02 | 70.73 |
| 100 | 33.33 | 28.89 | 34.69 | 31.43 | 31.34 |
| 150 | 25.00 | 21.05 | 19.64 | 22.67 | 27.27 |
| 200 | 6.10 | 4.88 | 8.82 | 4.92 | 5.26 |
| 250 | 2.50 | 3.23 | 2.50 | 5.41 | 3.45 |
| 300 | 0.00 | 0.00 | 1.61 | 0.00 | 1.89 |

Table S 4. Pollen germination rate under different PEG4000 concentration media

| Concentrations (g/L) | Germination rate (%) | | | | |
| --- | --- | --- | --- | --- | --- |
| 0 | 38.57 | 46.58 | 47.06 | 41.18 | 58.82 |
| 50 | 74.12 | 66.67 | 80.77 | 73.02 | 72.86 |
| 100 | 58.82 | 66.67 | 60.00 | 72.92 | 52.63 |
| 150 | 12.50 | 33.33 | 37.50 | 43.48 | 43.48 |
| 200 | 11.90 | 9.43 | 14.29 | 14.00 | 14.06 |
| 250 | 9.38 | 9.30 | 14.29 | 10.34 | 15.69 |
| 300 | 12.12 | 7.69 | 11.32 | 9.52 | 15.15 |

Table S 5. Pollen germination rate under different Boric acid concentration media

| Concentrations (mg/L) | Germination rate (%) | | | | |
| --- | --- | --- | --- | --- | --- |
| 0 | 7.55 | 14.29 | 6.45 | 9.52 | 12.33 |
| 100 | 65.12 | 64.29 | 60.00 | 72.97 | 59.09 |
| 200 | 80.85 | 71.43 | 69.23 | 69.12 | 78.43 |
| 300 | 59.52 | 66.67 | 61.22 | 68.42 | 63.64 |
| 400 | 51.35 | 46.88 | 45.71 | 39.62 | 54.69 |

Table S 6. Pollen germination rate under different Calcium nitrate concentration media

| Concentrations (mg/L) | Germination rate (%) | | | | |
| --- | --- | --- | --- | --- | --- |
| 0 | 8.57 | 11.76 | 6.25 | 6.45 | 6.25 |
| 100 | 53.45 | 60.00 | 67.57 | 60.61 | 66.67 |
| 200 | 71.43 | 65.91 | 77.55 | 72.41 | 71.88 |
| 300 | 78.38 | 81.25 | 85.96 | 79.59 | 73.17 |
| 400 | 51.35 | 57.14 | 76.60 | 62.50 | 60.53 |

Table S 7. Pollen germination after different temperature treatments

| Temperatures (°C) | Germination rate (%) | | | | |
| --- | --- | --- | --- | --- | --- |
| 15 | 20.27 | 26.67 | 25.00 | 24.24 | 17.14 |
| 20 | 36.11 | 33.33 | 31.91 | 33.33 | 32.69 |
| 25 | 76.92 | 71.43 | 71.79 | 70.21 | 80.36 |
| 30 | 84.38 | 70.59 | 77.33 | 71.70 | 75.00 |
| 35 | 36.36 | 27.78 | 26.92 | 41.30 | 38.78 |
| 40 | 3.08 | 6.25 | 9.76 | 1.59 | 6.67 |
| 45 | 2.94 | 1.96 | 6.90 | 6.56 | 5.26 |

Table S 8. Germination rate of different genotypes of pollen in optimised medium

| Genotypes | Germination rate (%) | | | | |
| --- | --- | --- | --- | --- | --- |
| Xuzishu 8 | 81.93 | 86.46 | 78.48 | 74.32 | 75.41 |
| Pushu 32 | 71.15 | 75.93 | 77.78 | 73.68 | 71.23 |
| Quanshu 830 | 80.77 | 73.33 | 76.19 | 78.05 | 82.35 |
| Yanshu 5 | 75.44 | 76.00 | 73.81 | 84.00 | 75.27 |
| Xushu 18 | 78.38 | 78.95 | 71.43 | 72.58 | 80.61 |
| Ningzishu 4 | 82.98 | 72.55 | 76.19 | 71.43 | 74.36 |
| Xushu 29 | 69.23 | 79.03 | 88.89 | 69.44 | 74.36 |
| Luoxushu 8 | 86.84 | 75.61 | 81.82 | 71.05 | 73.33 |
| Xuzihuaye | 83.05 | 77.61 | 78.85 | 79.51 | 78.76 |
| *I. batatas* (4*x*) | 44.44 | 45.45 | 42.86 | 51.02 | 61.90 |

Table S 9. Viability of pollen under different methods

| Methods | Pollen Viability (%) | | | | |
| --- | --- | --- | --- | --- | --- |
| TTC | 21.74 | 26.67 | 20.59 | 24.42 | 27.41 |
| Magenta acetate | 50.00 | 31.37 | 29.51 | 45.45 | 34.78 |
| FDA | 5.13 | 8.33 | 15.00 | 5.26 | 0.00 |
| *In vitro* germination | 81.93 | 86.46 | 78.48 | 74.32 | 75.41 |

Table S 10. Diameter of pollen grains (μm, excluding spines)

| 90.16 | 84.99 | 85.83 | 83.01 | 91.88 | 86.46 | 90.86 | 86.53 | 89.25 | 89.06 |
| --- | --- | --- | --- | --- | --- | --- | --- | --- | --- |
| 88.35 | 84.68 | 81.35 | 89.68 | 90.75 | 89.02 | 90.93 | 89.14 | 90.31 | 85.68 |
| 87.73 | 86.82 | 89.78 | 83.13 | 81.76 | 81.19 | 82.26 | 89.90 | 88.44 | 83.02 |

Table S 11. Length of spines (μm)

| 6.15 | 6.14 | 6.27 | 5.90 | 5.70 | 6.28 | 6.24 | 6.65 | 5.75 | 5.68 |
| --- | --- | --- | --- | --- | --- | --- | --- | --- | --- |
| 5.84 | 6.15 | 6.20 | 6.55 | 5.76 | 6.05 | 6.20 | 5.75 | 6.04 | 6.48 |
| 5.68 | 5.91 | 5.84 | 5.59 | 5.66 | 6.45 | 5.82 | 5.53 | 6.24 | 6.40 |

Table S 12. Diameter of pores (μm)

| 7.39 | 6.43 | 6.27 | 6.75 | 7.59 | 7.71 | 7.39 | 6.24 | 6.83 | 7.04 | 6.12 |
| --- | --- | --- | --- | --- | --- | --- | --- | --- | --- | --- |
| 6.12 | 6.27 | 5.82 | 7.11 | 7.45 | 6.70 | 6.90 | 6.75 | 6.27 | 6.35 | 6.12 |
| 6.56 | 6.72 | 6.40 | 7.60 | 6.79 | 6.91 | 6.91 | 7.92 | 7.21 | 5.92 | 6.56 |
